# Supplementary figures and images for: A semi-automated method for unbiased alveolar morphometry: Validation in a bronchopulmonary dysplasia model
Source: PLoS One. 2020 Sep 23;15(9):e0239562. doi: 10.1371/journal.pone.0239562 (PMC7511023; doi:10.1371/journal.pone.0239562)

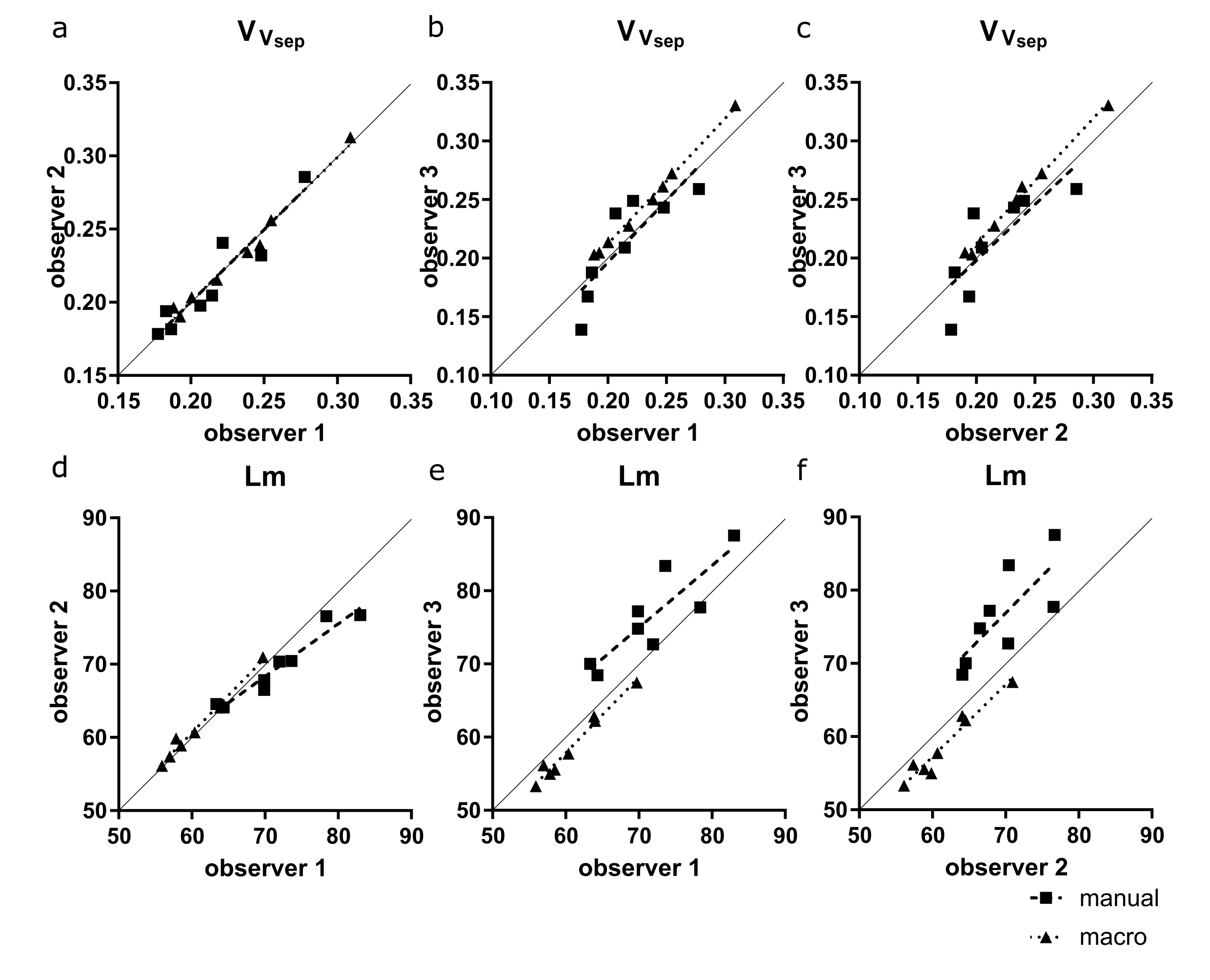

Supplement: S1 Fig — (TIF) [file pone.0239562.s001.tif]

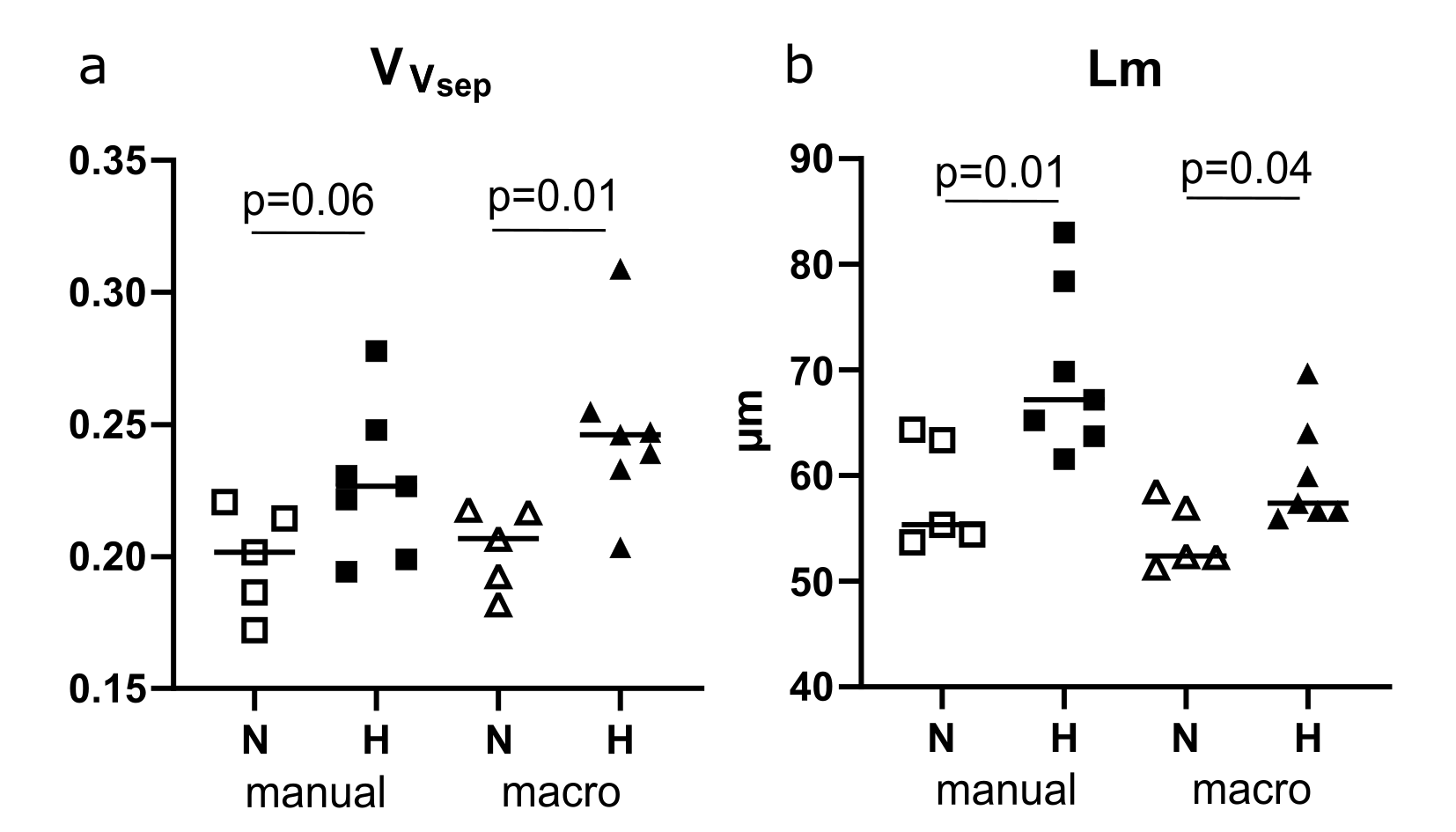

Supplement: S2 Fig — (TIF) [file pone.0239562.s002.tif]
