# Supplementary material for: A semi-automated method for unbiased alveolar morphometry: Validation in a bronchopulmonary dysplasia model
Source: PLoS One. 2020 Sep 23;15(9):e0239562. doi: 10.1371/journal.pone.0239562 (PMC7511023; doi:10.1371/journal.pone.0239562)
Supplement: S2 File — User manual of the morphometry_v4.0 plugin. (PDF) [file pone.0239562.s004.pdf]

USER MANUAL MORPHOMETRY_V4.0

Install FIJI

Download and install FIJI from https://imagej.net/Fiji/Downloads

Install the macro as a plugin

Download *morphometry_v4.0.ijm* from the supplement to this paper.

Open FIJI and go to *Plugins>Install…*

You are redirected to a search folder. Search for *morphometry_v4.0.ijm* and select it. Save it in the *plugins*-folder.

Restart FIJI

Preparation

Extract or picture square lung images (fields) from the studied lung slides. (How to do this depends on your histology workflow. Researchers using a slide scanner that exports in .czi-format, can use the random sampling algorithm that is available in a supplement to this paper.)

Save the required number of fields in .png-format together in 1 directory. Use sequential names: e.g. *field_1.png, field_2.png, …*

Important to note:

- Stain and process all lungs of an experiment in 1 batch in order to avoid differences in color intensity possibly affecting the segmentation of tissue in this plug-in.
- Select the required number of lung fields from the lung slice in a completely random way to avoid biasing your results.
- Make sure there are no subfolders or other files in the directory containing the lung fields, they will be overwritten.
- We recommend to use fields of 500µm by 500µm. For good quality segmentation, size of the fields should be at least 680 by 680 in pixels. Use an appropriate resolution.

Use of the plugin

Briefly, the plugin will iterate a two-step process for the manual selection of first non-parenchymal tissue (arteries, veins, airways) and then exudates. Counting is performed without manual input. For the first image in the folder, the plugin will request input to set the threshold. Below, we explain how to use the plugin step by step.

Open FIJI and start the morphometry-plugin: *Plugins>morphometry_v4.0*

Browse for the folder containing the lung fields you want to analyze and select it. Click *"Select"*.

The plugin will ask you to set the segmentation threshold by popping up the following dialog window.

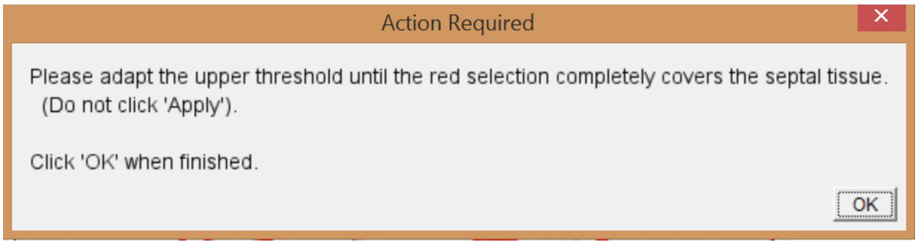

Use the second sliding bar in the separate *"Threshold"* window to segment the tissue from the air. Do NOT click *"Apply"* or any of the other commands in the *"Threshold"* window.

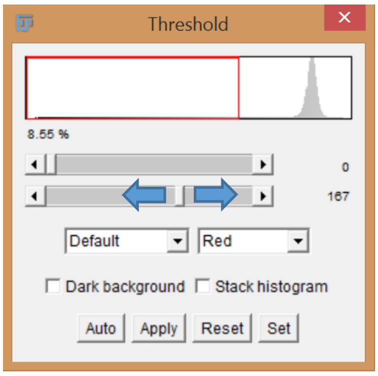

Make sure that all tissue connections are segmented. It is better to slightly overestimate the selection as the algorithm will clean out the noise in the alveolar air spaces.

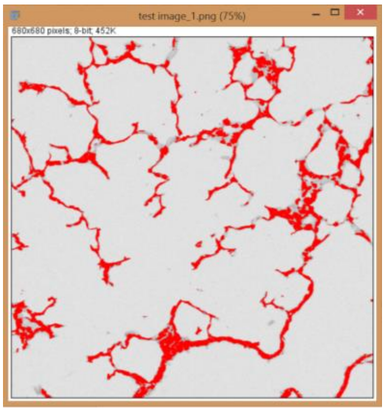

not all tissue segmented

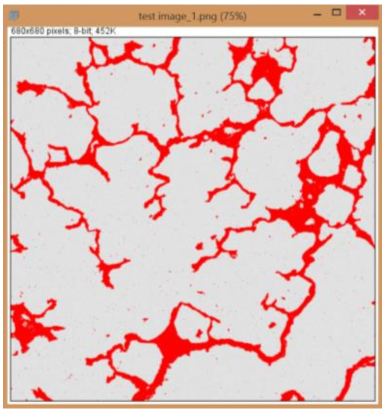

all tissue connections segmented little noise

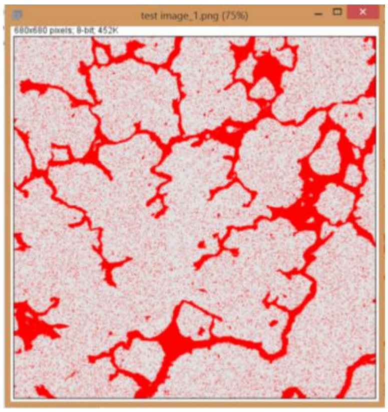

too much noise

Click *"OK"* in the dialog window when the threshold is set properly. The threshold is now set for all the fields in the folder, and you will not be asked again to set a threshold for every individual field. (Try to limit variation in staining intensity or contrast as much as possible. However, in our experience, minor differences do not bias the results too much due to the cleaning steps in the process creating the mask.)

The next step is to manually select areas to be excluded on every individual field. This is manually performed by adding non-parenchyma and afterwards exudate areas to a region of interest (ROI) which is excluded from the automatic analysis.

Several test images and several windows will open. Do not close any of them. Among them is a dialog window:

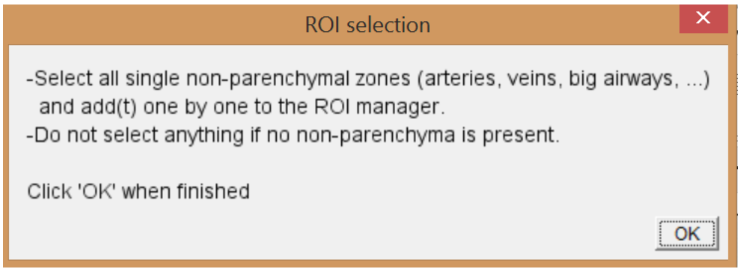

Manually delineate a non-parenchymal area (arteries, veins, big airways, …). Add it to the ROI manager by clicking *"Add(t)"* in the ROI manager or by hitting *"t"* on your keyboard. Delineate the next non-parenchymal area and add it to the ROI manager. Repeat until all non-parenchymal areas are in the ROI manager.

(Alternatively hold the *"shift"* button on your keyboard and select all non-parenchymal zones in 1 selection. Add this 1 selection at once to the ROI manager by clicking *"Add(t)"* in the ROI manager or by hitting *"t"* on your keyboard.)

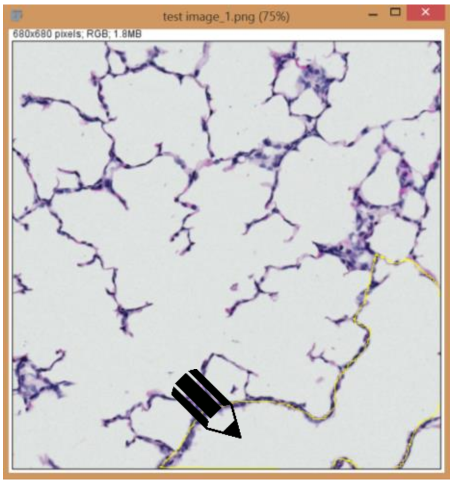

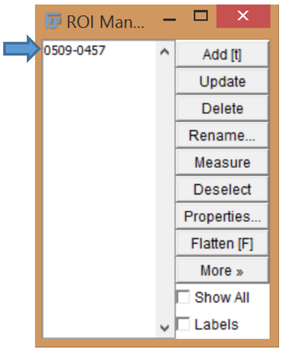

Click *"OK"* in the dialog window when all non-parenchymal areas are added to the ROI manager.

The plugin will process the image and open a new dialog window:

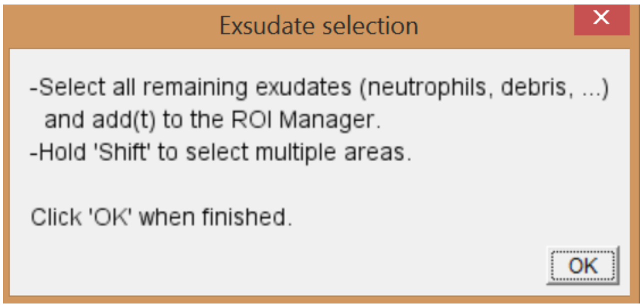

Select the remaining exudates on the processed image (non-parenchymal areas are filtered out and some exudates are automatically recognized). Exudates are neutrophils or debris in the alveolar airspaces that should not be counted as septal tissue. Hold the *"shift"* button on your keyboard to make multiple selections in 1 image. Add them all at once to the ROI manager by clicking *"Add(t)"* in the ROI manager or by hitting *"t"* on your keyboard.

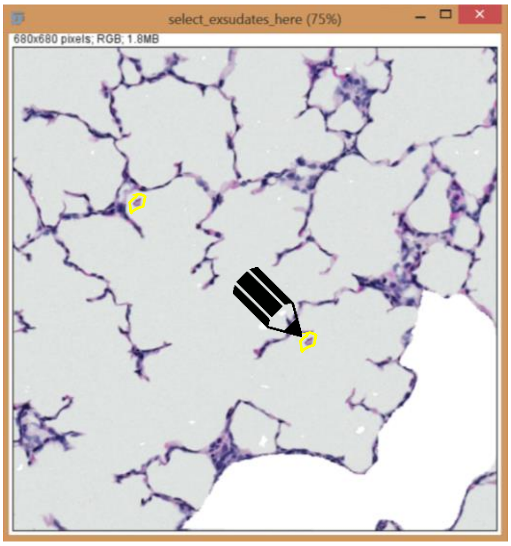

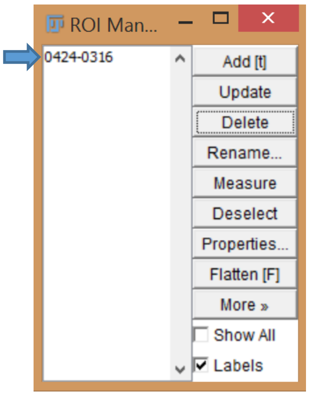

Click *"OK"* in the dialog window when all non-parenchymal areas are added to the ROI manager.

The plugin will now open up the following field in the directory. The plugin will iterate the process of delineating the non-parenchymal areas and selecting the exudates until all fields are done.

The counting itself is performed completely automatically. Counting results are saved in the window named *"Morphometry"*.

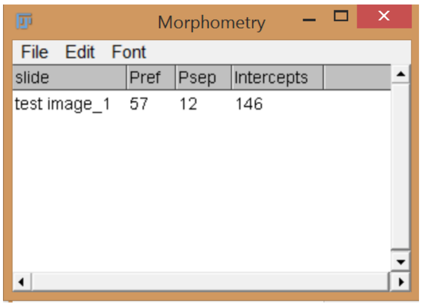

Plugin output

After analysis of all fields in the directory a *Morphometry.csv* file is saved with the counting results of all fields in the directory.

For each image the manual selections of non-parenchymal areas, the automatic selections of exudates and the manual selections of the remaining exudates are saved in .zip-folders. Also a resized version of the image (680x680 pixels) is saved in .bmp format, and an image of the tissue edges is saved in .tif format. These files can be used for re-analysis, or for a check of the accuracy of the segmentation.

All output files are saved in the directory containing the original images.

Morphometry.csv
test image_1.png
test image_1.zip
test image_1_edges.tif
test image_1_rescaled.bmp
test image_1autoexsudates.zip
test image_1manualexsudates.zip

Check the accuracy of the tissue recognition (optional)

In the supplement to this paper you can also find a plugin to visually check the segmentation of the septal tissue, taking into account thresholding, cleaning of the image, exclusion of exudates and non-parenchymal tissue. It is called *checkmorphometry_v4.0.ijm*. Install it and run it in the same way as the *morphometry_v4.0* plugin. It will ask you to select a folder. Select the directory containing the output of the *morphometry_v4.0* plugin. It will one by one open the fields in the folder and superpose the final septal tissue segmentation. By clicking *"OK"* in the dialog window it will move to the next field.

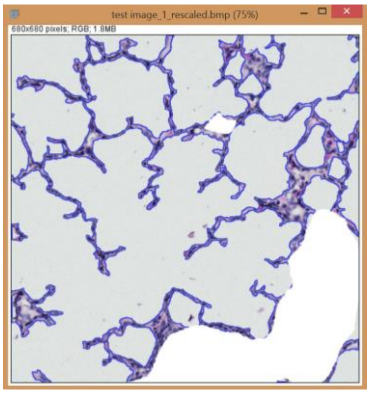

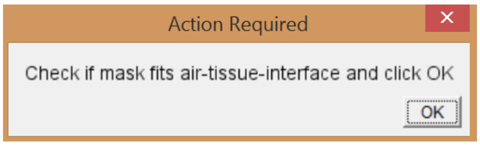

Calculate lung morphometry parameters (not included in plugin)

The plugin uses a quadratic test system of 64 points and 8 horizontal and 8 vertical lines with a total length of 9072pixels (unit length d = $\frac{9072}{64}$ = 141.75 pixels) on (rescaled) 680 by 680 pixel images.

- If 500µm by 500µm lung fields are used (rescaled to 680 by 680 pixel images in a resolution of 0.735µm/pixel) the unit length d = 104.2µm.
- If *x* µm by *x* µm lung fields are used (rescaled to 680 by 680 pixel images in a resolution of $\frac{x}{680}$ µm/pixel) the unit length d = $\frac{141.75*x}{680}\,\mu m.$

The test points falling on parenchymal (reference) tissue (P_{ref}), the points falling in alveolar septa (P_{sep}) and the intercepts with the test lines (I) are automatically counted. These numbers can be used to calculate various lung morphometry parameters.

The counts for P_{ref}, P_{sep} and I of all fields need to be summed up per lung. Using these summed counts, the volume density of alveolar septa ($V_{V_{sep}}$), the mean linear intercept of the airspaces (Lm), the mean transsectional wall length (Lmw) and the surface area density of the air spaces ($S_{V_{air}}$) of each lung can be calculated.

$$V_{V_{sep}} = \frac{\sum P_{sep}}{\sum P_{ref}}$$

$$Lm = 2 * d * \frac{\sum P_{ref} - \sum P_{sep}}{\sum I}$$

$$Lmw = 2 * d * \frac{\sum P_{sep}}{\sum I}$$

$$S_{V_{air}} = \frac{2 * \sum I}{d * \sum P_{sep}}$$

A calculation example is given below:

	A	B	C	D	E	F	G	H
1	*d =*	*104.2*	*µm*					
2								
3		**P_{ref}**	**P_{sep}**	**I**	**Vv_{sep} (%)**	**Lm (µm)**	**Lmw (µm)**	**Sv_{air} (µm²/µm³)**
4	**field 1**	64	13	156				
5	**field 2**	58	10	139				
6	**field 3**	61	14	148				
7	**...**							
8	**LUNG A**	183	37	443	0.20	68.68	17.41	0.23
9		*"=SUM(B4:B6)"*	*"=SUM(C4:C6)"*	*"=SUM(D4:D6)"*	*"=C8/B8"*	*"=2*B1*(B8-C8)/D8"*	*"=2*B1*C8/D8"*	*"=2*D8/(B1*C8)"*
10								

When the total lung volume is available, volume and surface density can be converted to absolute volume (V_{sep}) and surface (S) respectively. We refer to the guidelines for quantitative assessment of lung structure of the American Thoracic Society for more detailed information (1).

Trouble shooting (FAQ)

- Can I undo a step in the process if I for instance see that I made an incorrect selection?
  A wrong selection you want to remove before clicking "OK" can be deleted using the controls in the ROI manager window. Once you click "OK" in the dialog box and proceed it is impossible to go back and the only option is to add the field to a new folder and start again for the erroneous fields.
- The test grid does not seem to create correctly and the counts make no sense (0, 1, very high, …).
  Make sure the settings of your ImageJ (Edit>Options>…) are set as follows:
  - *Use inverting lookup table: false (under Appearance)*
  - *Foreground: white; background: black (under Colors)*
- Can I save my progress in the middle of a folder and continue later?
  There is no button to pause the analysis. Results are only saved automatically at the completion of all fields in the folder. We recommend not to put too many fields together in 1 folder for this reason. (We typically combine all fields of 1 lung in 1 folder.) If you need to pause before completing all fields in a folder, you can manually save the Morphometry *results window as a .csv file. Create a separate (sub)folder to analyze the remaining fields in a second run.*

1. *Hsia CC, Hyde DM, Ochs M, Weibel ER. An official research policy statement of the American Thoracic Society/European Respiratory Society: standards for quantitative assessment of lung structure. Am J Respir Crit Care Med. 2010;181(4):394-418.*
